# CRIF1 Deficiency Increased Homocysteine Production by Disrupting Dihydrofolate Reductase Expression in Vascular Endothelial Cells

**DOI:** 10.3390/antiox10111645

**Published:** 2021-10-20

**Authors:** Ikjun Lee, Shuyu Piao, Seonhee Kim, Harsha Nagar, Su-Jeong Choi, Byeong Hwa Jeon, Sang-Ha Oh, Kaikobad Irani, Cuk-Seong Kim

**Affiliations:** 1 Department of Physiology and Medical Science, College of Medicine, Chungnam National University, Daejeon 301-747, Korea; tw2622@gmail.com (I.L.); piaoshuyu@cnu.ac.kr (S.P.); wlxlsunny@naver.com (S.K.); harsha_nagar2002@yahoo.com (H.N.); 01030028473@naver.com (S.-J.C.); bhjeon@cnu.ac.kr (B.H.J.); 2 Department of Plastic and Reconstructive Surgery, College of Medicine, Chungnam National University, Daejeon 301-721, Korea; djplastic@cnu.ac.kr; 3 Department of Internal Medicine, Division of Cardiovascular Medicine, Carver College of Medicine, University of Iowa, Iowa City, IA 52242, USA; kaikobad-irani@uiowa.edu

**Keywords:** CR6 interacting factor 1, dihydrofolate reductase, homocysteine, folic acid

## Abstract

Elevated plasma homocysteine levels can induce vascular endothelial dysfunction; however, the mechanisms regulating homocysteine metabolism in impaired endothelial cells are currently unclear. In this study, we deleted the essential mitoribosomal gene CR6 interacting factor 1 (CRIF1) in human umbilical vein endothelial cells (HUVECs) and mice to induce endothelial cell dysfunction; then, we monitored homocysteine accumulation. We found that CRIF1 downregulation caused significant increases in intracellular and plasma concentrations of homocysteine, which were associated with decreased levels of folate cycle intermediates such as 5-methyltetrahydrofolate (MTHF) and tetrahydrofolate (THF). Moreover, dihydrofolate reductase (DHFR), a key enzyme in folate-mediated metabolism, exhibited impaired activity and decreased protein expression in CRIF1 knockdown endothelial cells. Supplementation with folic acid did not restore DHFR expression levels or MTHF and homocysteine concentrations in endothelial cells with a CRIF1 deletion or DHFR knockdown. However, the overexpression of DHFR in CRIF1 knockdown endothelial cells resulted in decreased accumulation of homocysteine. Taken together, our findings suggest that CRIF1-deleted endothelial cells accumulated more homocysteine, compared with control cells; this was primarily mediated by the disruption of DHFR expression.

## 1. Introduction

Homocysteine is a sulfur-containing amino acid and the precursor of the essential amino acid methionine. An elevated blood plasma homocysteine concentration, known as hyperhomocysteinemia, is an independent risk factor for vascular diseases (e.g., coronary artery, ischemic heart, and peripheral vascular diseases) [1,2]. Notably, mild and intermediate types of hyperhomocysteinemia are associated with various disorders including cardiovascular and neurodegenerative diseases [3,4,5]. Although there is growing evidence that elevated plasma homocysteine levels impair vascular function and induce vascular diseases [6], the mechanisms driving homocysteine metabolism have not yet been fully elucidated.

Homocysteine is mainly metabolized via remethylation or transsulfuration. In the remethylation pathway, homocysteine is converted into methionine by the addition of a methyl group from 5-methyltetrahydrofolate (MTHF) or betaine. MTHF is produced by the conversion of dietary folic acid (FA) into 5,10-methyltetrahydofolate, then into MTHF by the enzyme 5,10-methyltetrahydrofolate reductase (MTHFR). FA is the synthetic form of folate. Both folate and FA are metabolically inactive and must be reduced to participate in cellular metabolism [7]. Following absorption, FA is reduced to tetrahydrofolate (THF) by dihydrofolate reductase (DHFR). In the transsulfuration pathway, homocysteine is converted into cystathionine by cystathionine β-synthase, then into to cysteine using vitamin B6 as a cofactor. FA, which is a synthetic compound, cannot be metabolized unless it is reduced to dihydrofolate or THF [8,9]. High concentrations of unmetabolized FA may induce disorders of folate and methionine metabolism, leading to the formation of unmetabolized homocysteine, which acts as a pro-oxidant [10]. Increases in homocysteine levels can be caused by genetic defects in enzymes involved in homocysteine metabolism or by cofactor deficiencies [11]. Several recent studies have focused on examining the effects of homocysteine on endothelial injury; the overaccumulation of homocysteine is considered to be the primary mechanism driving hyperhomocysteinemia-related cardiovascular diseases [6,12]. Homocysteine is ubiquitous throughout the body; however, endothelial cells lining blood vessels may substantially contribute to plasma homocysteine concentrations [13]. To our knowledge, few studies have examined the production of homocysteine by injured endothelial cells.

CR6 interacting factor 1 (CRIF1) is a mitochondrial protein associated with large mitoribosomal subunits, and it is responsible for the production of polypeptides involved in oxidative phosphorylation and their insertion into the inner mitochondrial membrane [14]. Thus, CRIF1 is a potential target for mitochondrial dysfunction. CRIF1 deficiency impairs mitochondrial oxidative phosphorylation and reduces nitric oxide production while inhibiting tetrahydrobiopterin (BH4) biosynthesis enzymes such as guanosine triphosphate cyclohydrolase I, 6-pyruvoyltetrahydropterin synthase, sepiapterin reductase, and DHFR, leading to endothelial dysfunction [15]. DHFR regenerates BH4 from dihydrobiopterin and is an essential folate cycle enzyme required for homocysteine metabolism [16,17]. This study aimed to investigate the effects of CRIF1 downregulation on homocysteine accumulation and its molecular pathway.

## 2. Materials and Methods

### 2.1. Cell Culturing and Transfection

HUVECs at passage 2–9 were purchased from Clonetics (San Diego, CA, USA) and cultured in endothelial growth medium 2 at 37 °C with 5% CO_2_. For gene silencing, silencing RNA (siRNA) targeting CRIF1 (F: 5′-UGGAGGCCGAAGAACGCGAAUGGUA-3′; R: 5′-UACCAUUCGCGUUCUUCGGCCUCCA-3′), DHFR (Bioneer, Daejeon, South Korea), and non-coding siRNA (NC, Bioneer) were transfected into HUVECs or aortic segments using Lipofectamine 2000 (Invitrogen, Carlsbad, CA, USA) for 48 h, in accordance with the manufacturer’s instructions. Human and mouse MYC-DHFR and control plasmids (Sino Biological, Beijing, China) were transfected into HUVECs and aorta using Lipofectamine 2000 and then incubated for 24 h to induce gene overexpression.

### 2.2. Mouse Studies

All animal experiments were conducted at Chungnam National University (CNUH-019-A0016; approval date: 13 June 2019) in accordance with the guidelines of the Institutional Animal Care and Use Committee. Mice were maintained in a controlled environment (ambient temperature 22–24 °C; humidity 50–60%; 12 h light/dark cycle). Floxed CRIF1 (CRIF1flox/flox) mice were generated as described previously [14]. For generating Tek-CRIF1 mice, Tek-Cre transgenic mice (C57BL/6J) were purchased (Jackson Laboratory, Bar Harbor, ME, USA) and crossed with Floxed CRIF1 mice. Genotyping of the mouse strains were confirmed by polymerase chain reaction analysis of genomic DNA isolated from tail tissue using specific primers. The primers used for genotyping were as follows: CRIF1-Loxp (F: 5’-GGGCTGGTGAAATGTGTTG-3’, R: 5’-TCAGCTAGGGTGGGACAGA-3’) and Cre (F: 5’-GCGGTCTGGCAGTAAAAACTATC-3’, R: 5’-GTGAAACAGCATTGGTGTCACTT-3’). A DNA extraction kit (Toyobo, Iwakuni, Japan) was used in accordance with the manufacturer’s instructions. Mice were maintained in the animal facility in accordance with institutional guidelines.

### 2.3. Immunoblotting

HUVECs, mouse lung endothelial cells, and mouse aortic endothelial cells were homogenized using radioimmunoprecipitation assay buffer. After they were subjected to vortexing and sonication, the homogenized samples were centrifuged at 12,000× *g* for 15 min and the supernatant was harvested. Immunoblots were performed using 20 µg samples as described previously [15]. After electrophoresis and transferring to nitrocellulose membranes, blots were blocked using 5% skim milk over 1 h at room temperature. Specific primary antibodies for CRIF1 (sc-374122), DHFR (sc-377091), and β-actin (sc-47778) were purchased from Santa Cruz (Dallas, TX, USA). The membranes were incubated with 4000:1 of β-actin and 1000:1 of CRIF1 and DHFR, respectively, at 4 °C, overnight. Following TBST washing for 3 times, 4000:1 secondary antibody (Thermo-Fisher Scientific, Waltham, MA, USA) were treated to membrane for 1 h at room temperature. After incubation, membranes were washed 3 times and detected using the Super Signal Pico substrate by Thermo-Fisher Scientific.

### 2.4. Real-Time Quantitative Polymerase Chain Reaction

TRIzol reagent (Thermo Fisher Scientific, Waltham, MA, USA) was used to extract total RNA from HUVECs and lung endothelial cells in accordance with the manufacturer’s protocol. The primers used to perform real-time quantitative polymerase chain reaction on human serine hydroxymethyltransferase (SHMT) 1, SHMT2, and MTHFR were as follows: SHMT1 (F: 5’-CTGGCACAACCCCTCAAAGA-3’, R: 5’-AGGCAATCAGCTCCAATCCAA-3’), SHMT2 (F: 5’-GCCACGGCTCATCATAGCTG-3’, R: 5’-AGCAGGTGTGCTTTGACTTCA-3’), and MTHFR (F: 5’-CCGCACCATCATCCAGTACAT-3’, R: 5’-CCTCCGTTTCT CTCGCATTCT-3’). The primers used to detect mouse SHMT1, SHMT2, and MTHFR were as follows: SHMT1 (F: 5’-GGACAGTGATGCCGAGGTTTA-3’, R: 5’-TCGGTCCCGCCATAATACCTT-3’), SHMT2 (F: 5’-CCATCACAGCCAACAAGAACA-3’, R: 5’-TGGCTTGTCTCTGGGTCTTTG-3’), and MTHFR (F: 5’-CTGGGCACTGTTATCCATCCC-3’, R: 5’-TCCTGCTGATAGAGGGTGGC-3’). Amplification was performed using a Realplex thermocycler (Eppendorf, Hamburg, Germany). Relative expression was calculated in terms of fold change using the 2^−ΔΔCt^ equation. Data are expressed as means ± standard errors of the mean.

### 2.5. MTHF Content Measurement

Samples were prepared for and analyzed by high-performance liquid chromatography (HPLC) as described previously, with slight modifications [18,19]. Briefly, cells were harvested and resuspended in 57 mM ascorbic acid, then sonicated and vortexed. Following cell lysis, samples were incubated for 60 min at 37 °C, then diluted with potassium phosphate buffer (0.2 M potassium phosphate dibasic and 30 mM mercaptoethanol, pH 8.5) and heated for 10 min at 100 °C. Samples were centrifuged for 15 min at 10,000× *g*, and the supernatants were analyzed by HPLC. The MTHF content was quantified by measuring the fluorescence signal at excitation 295 nm, emission 360 nm. The mobile phase was methanol:0.6% acetic acid in water, 14:86 (*v*/*v*); the flow rate was set to 1 mL/min. MTHF standard was used to generate a calibration curve; standard samples were prepared with the protocol used for experimental sample preparation.

### 2.6. THF Content Measurement

To measure the THF content, harvested cells were resuspended in assay buffer (0.2 mM nicotinamide adenine dinucleotide phosphate, 1 mM dithiothreitol, 0.5 mM potassium chloride, 1 mM ethylenediaminetetraacetic acid, 20 mM sodium ascorbate, and 0.1 M potassium phosphate, pH 7.4) and incubated for 30 min at 37 °C, after which a stabilizing solution (0.2 g sodium ascorbate and 0.03 g dithiothreitol suspended in 1 mL distilled water) was added to samples. Then, the samples were sonicated and vortexed, followed by centrifugation for 15 min at 10,000× *g*. The supernatants were harvested and analyzed by HPLC using the mobile phase and flow rate described previously [19,20]. A fluorescence detector connected to the HPLC machine was used to quantify THF at excitation and emission wavelengths of 295 nm and 365 nm, respectively.

### 2.7. Ex Vivo Experiments

Aorta from 8–9 weeks old wild-type (WT) and CRIF1-deficient mice were harvested for the ex vivo experiments. After the mice were anesthetized, the descending thoracic aortae were carefully segregated from the mice and excess fat was removed. Then, the aortae were cut into 3–5-mm sections. Plasmid DNA or siRNA were transfected into the aortae with Lipofectamine 2000, following the same protocol used for HUVEC transfection. After transfection, the aortae were incubated in endothelial growth medium 2 at 37 °C.

### 2.8. Isolation of Mouse Endothelial Cells

Endothelial cells were isolated using lung tissue harvested from 8–10 week old mice or aortic segments by following the previous protocol [15]. The lungs excised from mouse or aortic pieces were placed in ice-cold phosphate-buffered saline solution and minced into small pieces. The minced tissue was incubated with collagenase I (Worthington, Lakewood, NJ, USA) at 37 °C and washed with phosphate-buffered saline; red blood cells were then removed using red blood cell lysis buffer. To isolate the endothelial cells, magnetic-activated cell sorting was performed in accordance with the manufacturer’s protocol (Miltenyi Biotec, Bergisch Gladbach, NRW, Germany); anti-CD31 and anti-CD45 antibodies attached to microbeads were used with magnetic-activated cell sorting to obtain CD45-negative and CD31-positive cells.

### 2.9. Immunofluorescent Staining

Immunofluorescence staining was performed following ex vivo transfection of aorta. The aorta was cut vertically and fixed in 4% paraformaldehyde for 24 h. Subsequently, they were permeabilized and blocked using 1% bovine serum albumin solution for 30 min. Anti-DHFR and anti-CD31 antibodies (Merck, Burlington, MA, USA) were added at 1:400 dilutions for 16 h; samples were then washed and incubated with 1:400 secondary antibody for 2 h. Images were obtained using an Axiophot microscope (Carl Zeiss, Oberkochen, BW, Germany).

### 2.10. Homocysteine Content Analysis

Homocysteine contents in HUVECs, growth medium, mouse plasma, and mouse endothelial cells were measured using a Homocysteine Fluorometric Assay Kit (Biovision, Milpitas, CA, USA). HUVECs or mouse endothelial cells were homogenized using assay buffer followed by sonication, and 50 μL of samples were used for analysis. For measuring homocysteine in growth medium or mouse plasma, 100 μL or 10 μL of sample were used respectively. Homocysteine was analyzed in accordance with the manufacturer’s instructions.

### 2.11. Statistical Analysis

Statistical analyses were performed using GraphPad Prism software (San Diego, CA, USA). The Student’s *t*-test was used to compare differences between two groups and to determine significance within the group versus to the cell without treatments. Data are presented as means ± standard errors of the mean. *p*-Values < 0.05 were considered to indicate statistical significance. All data are representative of at least three independent experiments.

## 3. Results

### 3.1. CRIF1 Knockdown Caused an Increase in Homocysteine Levels While Decreasing Folate Cycle Intermediates

**Figure 1:**
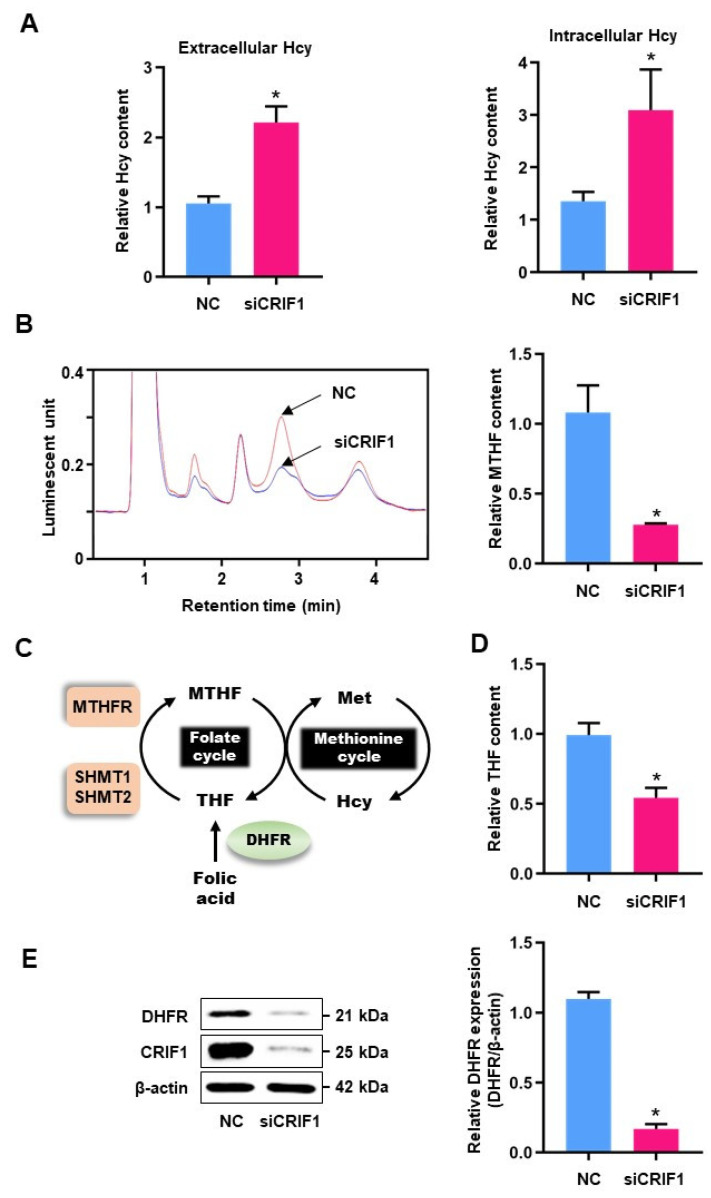
The levels of homocysteine, folate intermediates, and DHFR expression in CRIF1-deficienct HUVECs: (**A**) Hcy content in cells and supernatant media were measured using 100 pmol NC and siCRIF1-treated HUVECs. Relative quantitative analysis of Hcy content is shown as graph. (**B**) MTHF content of siCIRF1-treated HUVECs were analyzed using fluorescent-HPLC. Luminescent units of NC and siCRIF1-deleted HUVECs are shown as a graph (**left**), and the arrows indicate the peak of MTHF in cells. Relative quantification using peak-area is suggested as a graph (**right**). (**C**) Schematic model of the Hcy metabolism pathway via remethylation using MTHF synthesized through the FA cycle. (**D**) THF content was measured in NC and CRIF1 siRNA-treated cells. (**E**) DHFR protein expression was detected by immunoblotting. Protein level was quantified by relative densitometric assay. Data are presented with three independent experiments as the mean ± SEM. * *p* < 0.05 compared with NC siRNA-treated cells.

To investigate the effects of reducing CRIF1 expression on homocysteine accumulation, we examined homocysteine levels in cell extracts and culture supernatants. Homocysteine levels were higher in cells transfected with CRIF1 siRNA than in control cells (Figure 1A). Folate cycle intermediates contribute to homocysteine metabolism, especially MTHF, which plays an important role as a methyl group donor in folate metabolism [21]. Therefore, we measured MTHF content using HPLC coupled to a fluorescence detector. MTHF levels were significantly lower in CRIF1 siRNA-transfected HUVECs than in control cells (Figure 1B). MTHFR, SHMT1, and SHMT2 replenish cellular MTHF levels using various intermediates. THF is the first compound produced during the folate cycle (Figure 1C). We observed no significant differences in the mRNA expression levels of MTHFR, SHMT1, and SHMT2 between CRIF1 siRNA-transfected and control cells (Supplementary Materials Figure S1A); therefore, we examined the THF contents of CRIF1 knockdown cells. The levels of THF, a precursor of MTHF, were significantly lower in CRIF1 siRNA-treated cells than in NC-treated cells (Figure 1D). Next, we examined the expression of DHFR, a key enzyme in the folate cycle that is involved in MTHF and THF generation, in CRIF1 siRNA-treated and WT cells. DHFR expression levels were significantly lower in CRIF1 knockdown cells than in control cells (Figure 1E). These results suggest that CRIF1 downregulation caused an increase in homocysteine levels as well as decreases in folate cycle intermediate accumulation and DHFR expression.

### 3.2. CRIF1 Downregulation Impaired FA Metabolism in Endothelial Cells

**Figure 2:**
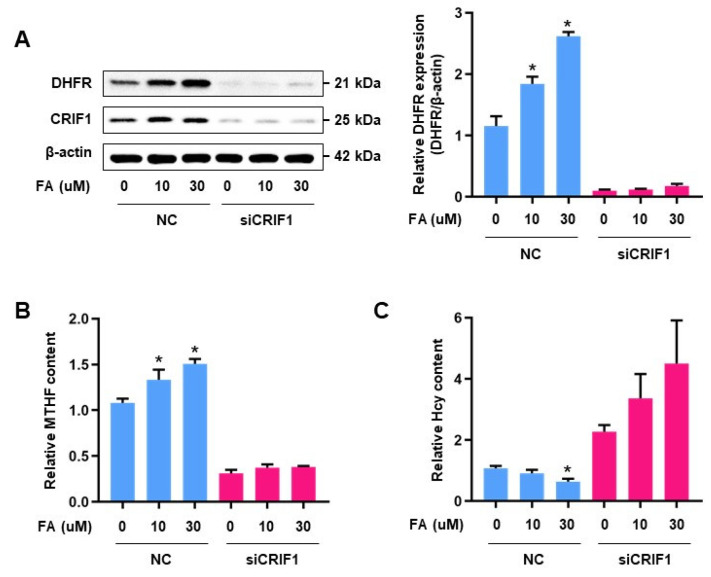
The effect of CRIF1 deficiency on FA-induced homocysteine and folate intermediates regulation: (**A**) DHFR protein level in HUVECS with 10 μM and 30 μM of FA after NC or CRIF1 siRNA treatments. Graph shows the densitometric assay of band intensity. (**B**) Fluorescent-HPLC analysis of MTHF content using the peak area of CRIF1-deleted HUVECs before FA treatments. (**C**) Relative quantification of the Hcy level with NC or siCRIF1 siRNA with/without FA. Data are presented with three independent experiments as the mean ± SEM. * *p* < 0.05 versus NC-treated cells with 0 μM FA in the NC group.

Under normal conditions, FA supplementation results in increased DHFR expression and decreased homocysteine levels [19,22]. Control cells exhibited increased DHFR expression in a concentration-dependent manner. However, in the CRIF1 knockdown cells, there were no significant changes in DHFR expression levels after FA treatment (Figure 2A). Furthermore, FA treatment had no effect on the MTHF content of CRIF1 knockdown cells (Figure 2B). Homocysteine levels were significantly lower in cells transfected with control siRNA that had been treated with 30 µM FA, compared with untreated cells transfected with control siRNA. However, FA supplementation did not reduce homocysteine levels in CRIF1 siRNA-treated cells (Figure 2C). These findings indicate that CRIF1 downregulation impaired FA metabolism in endothelial cells.

### 3.3. Increased Homocysteine Levels in CRIF1 siRNA-Treated HUVECs Were Mediated by DHFR

**Figure 3:**
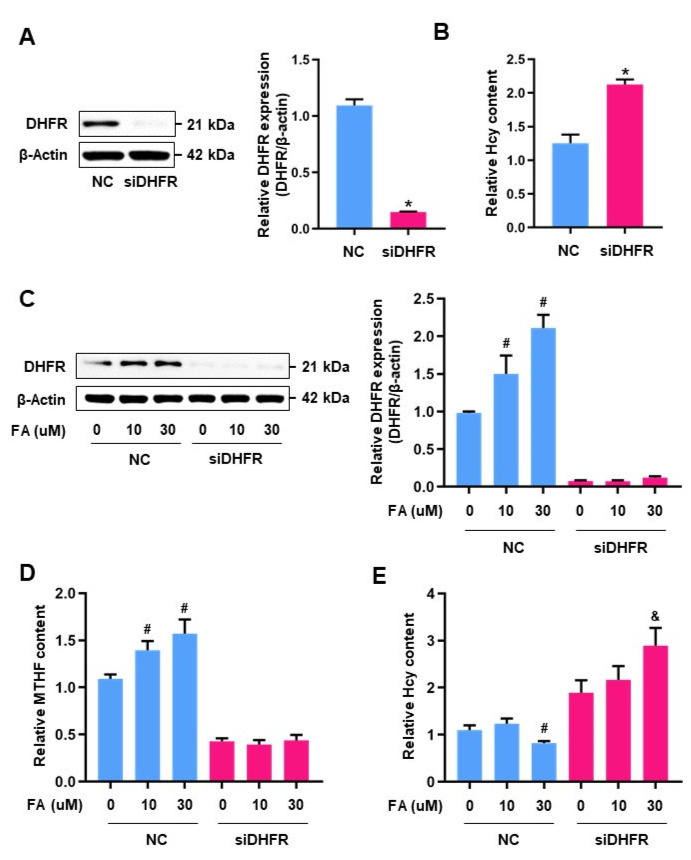
The effect of DHFR deficiency on homocysteine and folate intermediates in HUVECs: (**A**) Western blot analysis for DHFR expression in HUVECs after silencing DHFR by using 100 pmol siRNA over 48 h. The expression level of DHFR was quantified densitometrically. (**B**) The Hcy content of supernatant media of siDHFR-treated HUVECs was measured. Data are presented with three independent experiments as the mean ± SEM. * *p* < 0.05 compared with NC-treated cells. (**C**) HUVECs transfected with 100 pmol siDHFR before 10 μM or 30 μM FA treatment were used for measuring the protein level of DHFR. Densitometric analysis of DHFR expression is shown as a graph relatively. (**D**) Fluorescent-HPLC analysis of MTHF content using the. peak height of siDHFR-transfected HUVECs before FA treatments. (**E**) Relative quantification of the Hcy level with NC or DHFR siRNA with/without FA. Data are presented with three independent experiments as the mean ± SEM. # *p* < 0.05 versus NC-treated cells with 0 μM FA in the NC group, & *p* < 0.05 versus siDHFR-treated cells with 0 μM FA in the siDHFR group.

Inadequate FA intake is linked to abnormal plasma homocysteine concentrations, and FA fortification mediates a synergistic effect on homocysteine levels [22,23]. However, FA treatment did not affect DHFR expression or MTHF and homocysteine levels in CRIF1 siRNA-treated cells (Figure 2A). DHFR is the gateway enzyme for FA to enter the folate metabolic pathway, and DHFR did not exhibit any significant changes in expression following FA treatment; therefore, we hypothesized that the impairment of DHFR in CRIF1 knockdown cells may have caused the increase in homocysteine levels. To investigate the role of DHFR in the folate cycle, we downregulated DHFR via siRNA transfection (Figure 3A) and examined the effects on homocysteine and MTHF levels. Homocysteine levels were significantly higher in DHFR siRNA-transfected cells than in cells transfected with control siRNA (Figure 3B).

Next, we investigated the effects of DHFR downregulation on FA-triggered DHFR expression as well as MTHF and homocysteine biosynthesis. HUVECs were transfected with DHFR siRNA for 24 h, then treated with FA for another 24 h. In cells transfected with control siRNA, DHFR expression increased following FA treatment; cells transfected with DHFR siRNA exhibited no differences in DHFR expression, regardless of FA treatment status (Figure 3C). Moreover, DHFR knockdown suppressed FA-induced MTHF synthesis (Figure 3D) and homocysteine alleviation (Figure 3E).

### 3.4. DHFR Inhibited the Levels of Homocysteine in CRIF1-Deficienct Endothelial Cells

**Figure 4:**
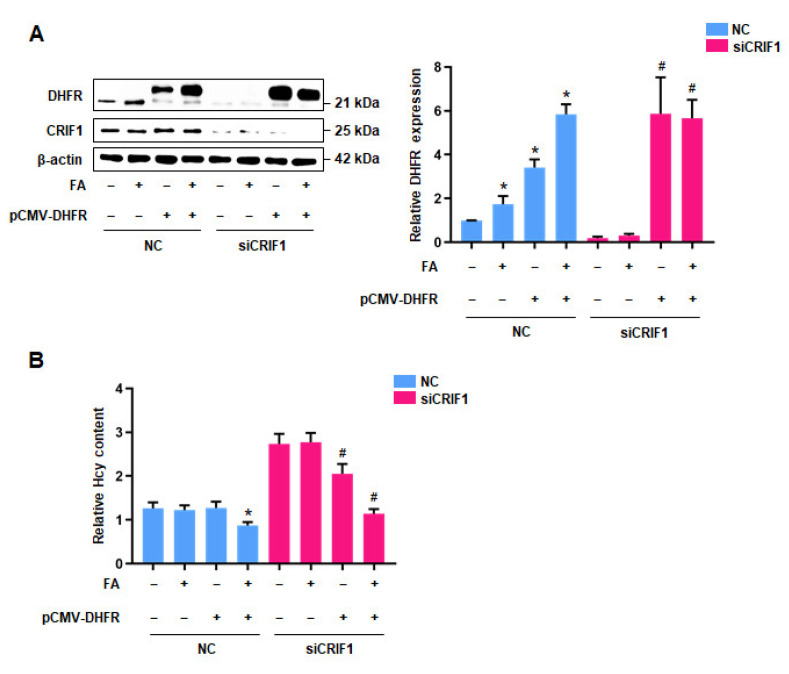
The effect of DHFR overexpression on Hcy regulation in HUVECs: (**A**) Western blot analysis for DHFR protein level using NC or siRNA-transfected HUVECs followed by pCMV-DHFR and FA treatments. (**B**) Relative quantification of the Hcy level of FA and pCMV-DHFR treatments after CRIF1 siRNA transfection. Data are presented with three independent experiments as the mean ± SEM. * *p* < 0.05 versus NC-treated cells without FA and pCMV-DHFR in the NC group, # *p* < 0.05 versus siCRIF1-treated cells without FA and pCMV-DHFR in the siCRIF1 group.

Because DHFR plays an important role in FA-induced MTHF synthesis and homocysteine regulation, we investigated the effects of plasmid-mediated DHFR overexpression on homocysteine accumulation in CRIF1 siRNA-transfected cells. A DHFR overexpression vector was transfected into control and CRIF1 knockdown cells; DHFR expression was significantly higher in plasmid-transfected cells than in control cells (Figure 4A). Furthermore, homocysteine levels were lowest in CRIF1 knockdown cells that had been transfected with the DHFR overexpression vector and treated with FA; this effect was stronger in CRIF1 knockdown cells than in CRIF1 siRNA-transfected cells and in cells co-transfected with the DHFR overexpression vector (Figure 4B). These findings suggest that DHFR downregulation is likely to have caused the increase in homocysteine levels in CRIF1 knockdown cells; moreover, DHFR plays an important role in regulating homocysteine via folate metabolites.

### 3.5. CRIF1 Deletion Caused an Elevation in Homocysteine Levels and a Decrease in Folate Metabolites In Vivo

**Figure 5:**
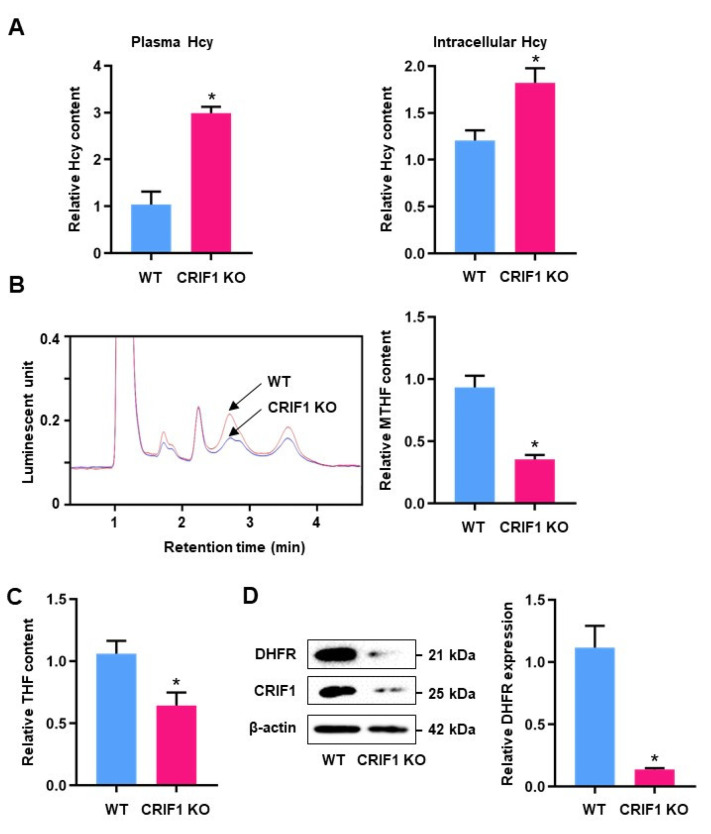
The levels of homocysteine, MTHF, THF, and DHFR expression in mouse endothelial cells. (**A**) Hcy content in lung endothelial cells and plasma from WT and CRIF1 KO mouse were measured. Relative quantitative analysis of the Hcy content is shown as a graph. (**B**) MTHF content of WT and KO endothelial cells were quantified by fluorescent-HPLC. Arrows on the luminescent signal indicate an MTHF peak of WT and CRIF1 KO endothelial cells, and the peak-height-based quantification of MTHF is shown as a graph (right). (**C**) The THF level of endothelial cells isolated from WT and CRIF1 KO mouse was quantified and is shown as a graph. (**D**) Western blot analysis of DHFR expression of WT and KO mouse endothelial cells. DHFR level was quantified densitometrically. Data are presented with three independent experiments as the mean ± SEM. * *p* < 0.05 compared with WT group.

Because homocysteine levels increased in the CRIF1-silenced endothelial cells due to the inhibition of DHFR, we examined the homocysteine and MTHF levels in endothelial cells from CRIF1 KO mice. CRIF1 KO mice exhibited significantly higher homocysteine levels in both plasma and endothelial cells compared to WT mice (Figure 5A). Next, we measured MTHF and THF levels in endothelial cells from CRIF1 KO mice via fluorescence HPLC. The MTHF and THF contents were markedly lower in endothelial cells from CRIF1 KO mice, compared with cells from WT mice (Figure 5B,C). No significant differences in the expression levels of enzymes involved in THF and MTHF biosynthesis were present (Supplementary Materials Figure S1B). Furthermore, DHFR expression levels were lower in lung endothelial cells from CRIF1 KO mice compared with cells from control mice (Figure 5D). Taken together, our results suggest that the downregulation of CRIF1 in mouse endothelial cells caused an increase in homocysteine levels and led to the disruption of folate metabolism.

### 3.6. Elevated Homocysteine Concentrations Were Mediated by DHFR in CRIF1 KO Mice

**Figure 6:**
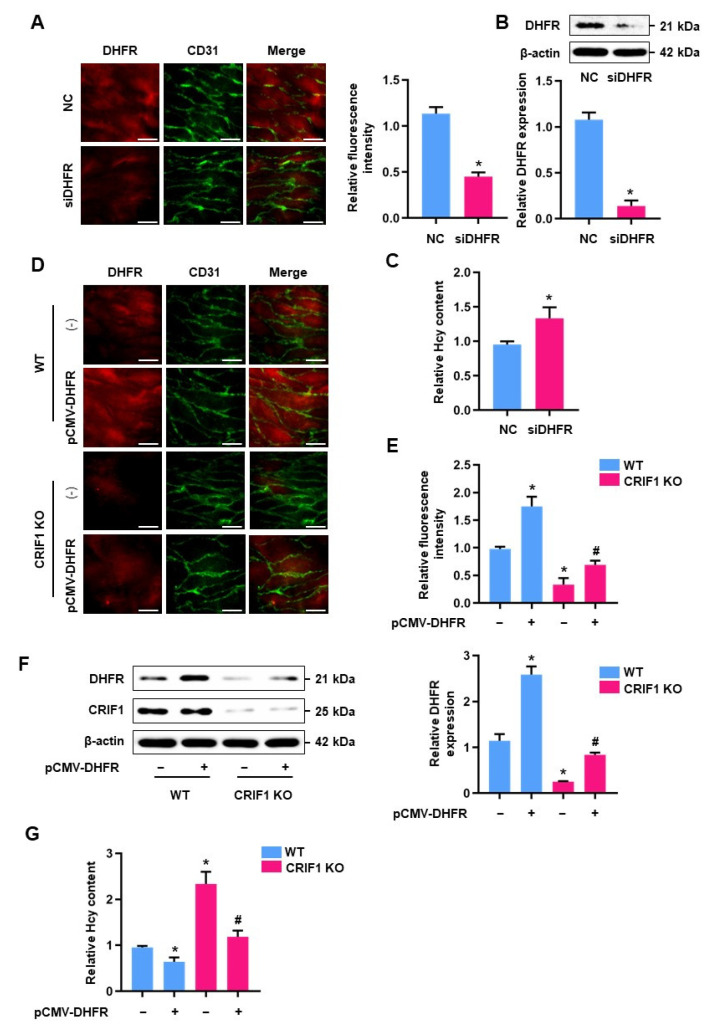
The effect of DHFR overexpression on homocysteine and folate intermediates in mouse endothelial cells: (**A**) Immunofluorescence staining of DHFR (red) and CD31 (green) in the NC or siDHFR-transfected aortic endothelial surface. The scale bar indicates 10 μm. Quantification of fluorescence signal was normalized to the NC group (right). (**B**) DHFR expression of ex vivo transfection in mouse aortic endothelium. Immunoblotting analysis for quantifying the DHFR level is shown as a graph. (**C**) Relative quantification of the Hcy content in aortic endothelial cells after ex vivo transfection of siDHFR. (**D**,**E**) DHFR protein levels of WT and CRIF1 KO mouse in aortic endothelium were analyzed by immunofluorescent staining after ex vivo transfection of pCMV-DHFR, and the scale bar indicates 10 μm. Signal intensity was quantified and suggested as a graph. (**F**) Immunoblotting assay of ex vivo-induced DHFR transfection on aorta. Quantitative analysis of DHFR level is suggested as a graph, relatively. (**G**) The relative Hcy content of pCMV-DHFR treatments on aortic endothelium. Data are presented with three independent experiments as the mean ± SEM. * *p* < 0.05 compared with WT mice; # *p* < 0.05 compared with CRIF1 KO mice.

To explore the role of DHFR in the regulation of homocysteine accumulation, we employed an ex vivo mouse aortic segments endothelium model. Aorta isolated from WT mice was transfected with DHFR siRNA for 24 h; en face staining was performed to investigate DHFR expression. DHFR fluorescence intensity was significantly lower in DHFR siRNA-transfected aorta compared with the control group (Figure 6A). Western blot analyses also revealed a similar pattern in DHFR expression in aortic endothelial cells (Figure 6B). Next, we investigated the effects of DHFR downregulation on homocysteine accumulation using the ex vivo model. Reducing DHFR expression caused an increase in homocysteine levels in aortic endothelial cells (Figure 6C). To investigate the effects of DHFR overexpression on homocysteine levels in mice endothelial cells, en face-prepared mouse aortae were transfected with a DHFR overexpression vector, then subjected to DHFR and CD31 staining (Figure 6D). DHFR fluorescence signals that co-localized with CD31 were higher in the DHFR overexpression group, while co-staining was only weakly detected in the CRIF1 KO group. DHFR expression was partially recovered in CRIF1 KO mice transfected with the DHFR overexpression vector (Figure 6E); a similar phenomenon was observed in endothelial cells isolated from CRIF1 KO mice that had been transfected with the DHFR overexpression vector (Figure 6F). Furthermore, homocysteine levels were markedly lower in aortic endothelial cells from CRIF1 KO mice transfected with the DHFR overexpression vector, compared with cells that lacked DHFR overexpression (Figure 6G). These data indicate that DHFR downregulation promoted homocysteine accumulation, and the increase in homocysteine levels observed in CRIF1 KO lines was closely related to DHFR expression in aortic endothelial cells.

## 4. Discussion

Elevated plasma homocysteine levels are a risk factor for vascular diseases as well as some cognitive impairments [1,4,24]. However, researchers have not yet investigated the relationship between damaged endothelial cells and high homocysteine concentrations. In the present study, we found that CRIF1 downregulation in endothelial cells induced a significant increase in homocysteine levels; this was associated with a decrease in the levels of the folate cycle intermediates THF and MTHF, which was mediated by the downregulation of DHFR. FA supplementation was unable to reinstate homeostatic concentrations of homocysteine and folate cycle intermediates. These findings provide important insights into the role of DHFR in homocysteine metabolism in CRIF1-deficient endothelial cells.

Increased homocysteine uptake contributes to various cytopathic effects in neuronal and vascular cells [25,26]. However, few studies have investigated homocysteine metabolism in various human cell types. Homocysteine is produced in various cells including proliferating immune-competent cells and endothelial cells [27,28,29]. Moreover, Sharma et al. found that homocysteine is generated in all cell types [30]. Endothelial cells can accumulate homocysteine to high intracellular concentrations, in contrast to the plasma homocysteine concentration gradient [31]. In this study, we induced CRIF1 downregulation, then observed intracellular and extracellular increases in homocysteine levels. This suggests that the endothelial cells were both impaired by elevated plasma homocysteine levels and generated increased levels of intracellular homocysteine.

Homocysteine is primarily metabolized by the folate pathway and the methylation pathway [32]. In most tissues, high levels of homocysteine are associated with decreased methylation potential [33,34,35]; however, FA supplementation contributes to methionine synthesis, which is associated with reduced homocysteine levels [34,36]. Hyperhomocysteinemia is associated with reduced MTHFR expression levels as well as the inhibition of homocysteine methyltransferase activity [37,38]. In this study, we examined the mRNA expression levels of MTHFR, SHMT1, and SHMT2 in CRIF1 siRNA-transfected cells. While CRIF1 siRNA transfection did not have a significant effect on SHMT1, SHMT2, or MTHFR mRNA expression levels (Supplementary Materials Figure S1), it reduced the levels of the folate cycle intermediates THF and MTHF.

THF is the major form of FA, which transfers its one-carbon group to the methylation pathway; THF is also involved in the regulation of dietary folate metabolism and the maintenance of the intracellular folate pool [39]. MTHF is a circulating metabolite of FA that contributes to homocysteine metabolism. To investigate whether reduced THF and MTHF concentrations could be recovered, dysfunctional endothelial cells transfected with CRIF1 siRNA were supplemented with FA.

FA deficiency causes depletion of the folate pool and contributes to elevated homocysteine levels. This is because folate metabolism regulates homocysteine remethylation with MTHF, which transfers its methyl group to homocysteine. THF is not directly involved in homocysteine remethylation; it receives one-carbon units from other molecules and transfers them to the methylation pathway [39]. FA supplementation leads to elevated THF and MTHF levels, which are associated with decreased homocysteine concentrations [36,40]. Moreover, in human intestinal microvascular endothelial cells and cytokine-stimulated cells, FA treatment causes a reduction in homocysteine concentration [41,42]. However, in endothelial cells transfected with CRIF1 siRNA, we observed no significant differences in THF or MTHF levels, regardless of FA treatment. This suggests that because of the low THF and MTHF levels, less FA was transported to the folate cycle, thus causing an elevation in homocysteine levels.

In addition to the effects of reduced homocysteine concentrations, the cardiovascular benefits of FA supplementation may be related to the enhanced biosynthesis of glutathione and other antioxidative markers [43]. An important finding of this study was that the FA cycle was impaired in CRIF1-deleted endothelial cells, causing FA to lose its homocysteine-lowering effect. Therefore, the mechanism underlying the dysfunction of the FA uptake system should be explored further, along with the effects of FA treatment on homocysteine accumulation in CRIF1-deficient endothelial cells.

DHFR plays a critical role in preserving the folate pool by reducing intermediates using nicotinamide adenine dinucleotide phosphate as a cofactor, in combination with enhancement of BH4 and nitric oxide bioavailability [20]. In our previous study, we found that scavenging mitochondrial reactive oxygen species partly recovered the expression levels of BH4 synthesis enzymes including guanosine triphosphate cyclohydrolase I, pyruvoyltetrahydropterin synthase, and sepiapterin reductase; it did not alter DHFR expression [15]. Taken together, our results suggest that downregulation of DHFR prevented the entry of FA into the folate cycle, thus promoting the accumulation of homocysteine in CRIF1 knockdown cells.

To assess our hypothesis in vitro and in vivo, we examined THF, MTHF, and homocysteine levels following the knockdown and overexpression of DHFR in CRIF1 knockdown cells. We found that the expression levels of DHFR were higher in endothelial cells treated with FA than in untreated cells, consistent with the results of previous studies. In CRIF1 knockdown and DHFR knockdown endothelial cells, FA supplementation did not have an effect on MTHF restoration and homocysteine alleviation. DHFR overexpression recovered the impaired homocysteine homeostasis in CRIF1 knockdown cells, which indicated that DHFR downregulation may be a major factor involved in homocysteine dysregulation in CRIF1 siRNA-transfected endothelial cells.

Although FA has been used in the treatment of high homocysteine levels for many years, controversies remain regarding the preventative effects of FA against cardiovascular diseases. Some studies reported that FA supplementation did not significantly decrease homocysteine levels or the risk of death from cardiovascular diseases [38,44]. Previously, we generated endothelial CRIF1 KO cells and mouse models to emulate endothelial dysfunction [45]. Although CRIF1 downregulation could not fully mimic in vivo endothelial dysfunction, our results highlight a possible pathophysiological mechanism to explain the ineffectiveness of FA treatment in lowering homocysteine levels.

## 5. Conclusions

In summary, our findings suggest that homocysteine production increased in CRIF1-deficient endothelial cells because of reduced DHFR expression and impaired folate metabolism. Therefore, DHFR offers a potential therapeutic target for lowering homocysteine concentrations in homocysteine-related diseases.

## Data Availability

The data is available within the article and supplementary materials.

## Supplementary Materials

The following are available online at www.mdpi.com/article/10.3390/antiox10111645/s1, Figure S1A: Real time PCR analysis of SHMT1, SHMT2 and MTHFR mRNA expression in HUVECs, Figure S1B: Quantitative mRNA assay of SHMT1, SHMT2 and MTHFR gene in mouse lung endothelial cells.
