# Supplementary material for: CRIF1 Deficiency Increased Homocysteine Production by Disrupting Dihydrofolate Reductase Expression in Vascular Endothelial Cells
Source: Antioxidants (Basel). 2021 Oct 20;10(11):1645. doi: 10.3390/antiox10111645 (PMC8614757; doi:10.3390/antiox10111645)
Supplement: Supplementary file 1 [file antioxidants-10-01645-s001.zip › antioxidants-1412379-supplementary.pdf]

Supplementary Figure S1.

A.

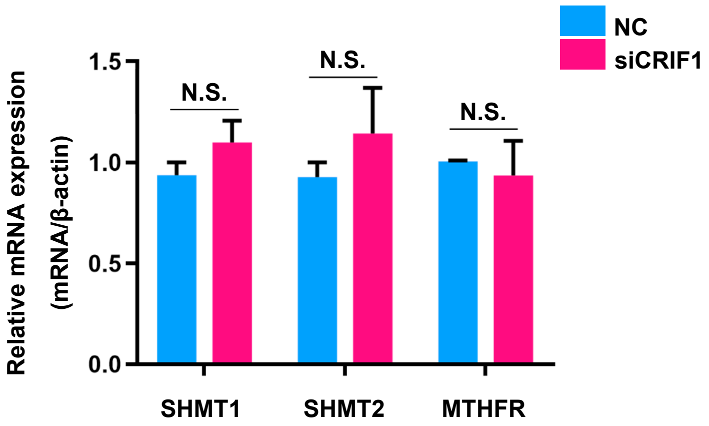

B.

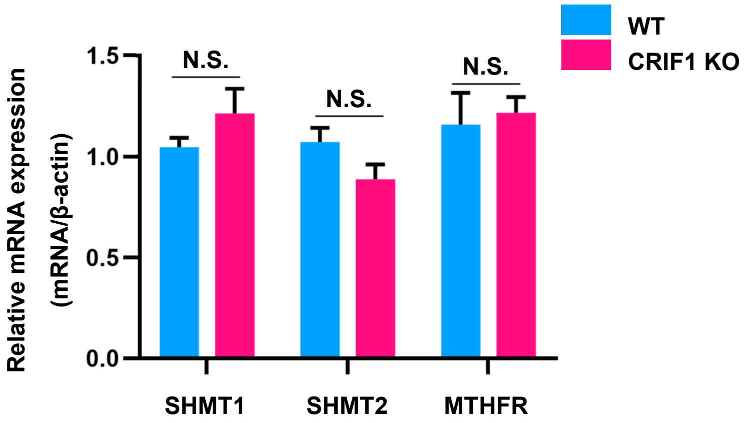

Figure S1. (A) Real time PCR analysis of SHMT1, SHMT2 and MTHFR mRNA expression. Expression was normalized to NC siRNA group and GAPDH was used for internal control. Data are presented with three independent experiments as the mean ± SEM. *$p < 0.05$ compared with NC siRNA treated cells. **(B)** Quantitive mRNA assay of SHMT1, SHMT2 and MTHFR gene in mouse lung endothelial cells. Gene expression was normalized to WT mouse group by mRNA expression. Data are presented with three independent experiments as the mean ± SEM. *$P < 0.05$ compared with WT mice.
